# Noise-Driven Stem Cell and Progenitor Population Dynamics

**DOI:** 10.1371/journal.pone.0002922

**Published:** 2008-08-13

**Authors:** Martin Hoffmann, Hannah H. Chang, Sui Huang, Donald E. Ingber, Markus Loeffler, Joerg Galle

**Affiliations:** 1 Interdisciplinary Centre for Bioinformatics, University of Leipzig, Leipzig, Germany; 2 Vascular Biology Program, Department of Pathology and Surgery, Children's Hospital and Harvard Medical School, Boston, Massachusetts, United States of America; 3 Program in Biophysics, Harvard University, Boston, Massachusetts, United States of America; 4 MD-PhD Program, Harvard Medical School, Boston, Massachusetts, United States of America; 5 Institute for Biocomplexity and Informatics, University of Calgary, Calgary, Canada; 6 Institute for Medical Informatics, Statistics, and Epidemiology, University of Leipzig, Leipzig, Germany; University of Arizona, United States of America

## Abstract

**Background:**

The balance between maintenance of the stem cell state and terminal differentiation is influenced by the cellular environment. The switching between these states has long been understood as a transition between attractor states of a molecular network. Herein, stochastic fluctuations are either suppressed or can trigger the transition, but they do not actually determine the attractor states.

**Methodology/Principal Findings:**

We present a novel mathematical concept in which stem cell and progenitor population dynamics are described as a probabilistic process that arises from cell proliferation and small fluctuations in the state of differentiation. These state fluctuations reflect random transitions between different activation patterns of the underlying regulatory network. Importantly, the associated noise amplitudes are state-dependent and set by the environment. Their variability determines the attractor states, and thus actually governs population dynamics. This model quantitatively reproduces the observed dynamics of differentiation and dedifferentiation in promyelocytic precursor cells.

**Conclusions/Significance:**

Consequently, state-specific noise modulation by external signals can be instrumental in controlling stem cell and progenitor population dynamics. We propose follow-up experiments for quantifying the imprinting influence of the environment on cellular noise regulation.

## Introduction

A growing body of evidence indicates that noise is not generally detrimental to biological systems but can be employed to generate genotypic, phenotypic, and behavioral diversity [1]–[5]. In particular, noise-driven solutions are expected to prevail in cellular adaptation to variable environments [6]. It has been proposed that biological systems have built-in molecular devices for noise control [7]–[12]. These mechanisms are of specific importance in developing organisms [11], [13]. This view is supported by experimental findings demonstrating that noise is down-regulated in embryonic stem cells [14], [15] and that fluctuations of the transcription factor Nanog predispose these cells towards differentiation [16]. The results of the present study suggest that noise regulation can be an effective strategy in stem cell differentiation.

Stem cells are characterized by their ability to self-maintain and generate differentiated cell types and functional tissues. Moreover, they show flexibility and reversibility in their use of these options [17], [18]. Populations derived from these cells, subsequently denoted as ‘stem cell populations’, comprise stem cells, progenitors, and differentiated cells. Their population structure is strongly influenced by environmental factors such as specific cell-cell interactions [19], growth factor and oxygen supply [20], as well as the geometry and mechanical properties of the local environment [21]–[23]. Changing these factors results in either cell death or adaptation within days [24]–[28]. Recently, progress has been made in the modeling and understanding of these processes on different levels of complexity [3], [29]–[36].

Our previous studies on stem cell population dynamics focused on the reversibility and stochasticity of cellular fate decisions [37], [38]. In the model of Roeder et al. [35], [36], [38] individual cells gain and loose stem cell properties depending on whether they localize inside or outside a specific niche environment, respectively. Thus, the environment directs the cellular fate and the reversibility of cell fate decisions is enabled by probabilistic switches between different micro-environments. The model well described several experimental data sets on the *in vivo* organization of normal and malignant hematopoietic stem cell populations [35], [36], [39]. However, even within homogeneous *in vitro* environments stem cells are capable of expanding and maintaining the aforementioned stem cell populations. For modeling these systems the present study expanded the ideas of Roeder et al. [35], [36] by assuming that cells gain and loose stem cell properties according to a probabilistic process whose state-specific amplitudes are set by the environment. Within this approach cell fate decisions are basically reversible. The assumed cell state fluctuations can be hypothesized to be generated by intra- and extracellular noise triggering random transitions between different regulatory network activation patterns. This concept is in agreement with experimental findings demonstrating that epigenetic gene silencing, known to be instrumental in cell differentiation and fate control, has a strong stochastic component [40], [41].

The regularity of biological development in spite of the ubiquitous presence of noise has raised the concept of a ‘potential energy landscape’ or ‘attractor landscape’ explaining cell differentiation and phenotypic diversification in terms of non-linear systems theory and non-equilibrium thermodynamics [22], [42]–[44]. In this concept, cells visit their accessible states driven by differences in potential energy and non-state-specific, so-called additive noise. Potential minima constitute attractor states corresponding to population density maxima in steady state. The alternative concept put forward in the present study assumes that noise is predominant in most cellular states. Its essence is that the population density is determined by state-specific, so-called multiplicative noise forming a ‘noise landscape’, with low noise states representing the attractor states. Cells subjected to an environment not matching their internal state are assumed to be destabilized by a high noise amplitude. They subsequently adapt to this environment by traveling towards low noise states [34].

Recently, we have studied biochemically induced differentiation and dedifferentiation in promyelocytic precursor cells by measuring the inducer dose-dependent dynamics of cell differentiation as observed by the expression of a specific cell surface marker [27]. Results from our model agreed well with the experimental data, thus demonstrating the utility of our alternative description. This suggests that stem cell and progenitor population dynamics can be effectively driven by state-specific noise, thereby providing a new vista onto phenomena like stem cell maintenance, plasticity, and environmental adaptation. We propose follow-up experiments for quantifying environmental influence on cellular noise regulation.

## Results

In the following paragraphs we describe our model and illustrate its general behavior for different parameter settings. Subsequently, the model is applied to a set of experimental data.

### Model

The present study focuses on the degree of differentiation as the basic cellular attribute of interest. It is defined as the loss of stem cell properties and goes along with but is not identical to lineage commitment. Cell differentiation is quantified by a variable *α* taking values between zero (full stem cell potential) and one (complete cell differentiation). Each value of *α* may stand for a set of regulatory network activation patterns. The overabundance of these patterns as seen in gene expression profiles [30] suggests the use of a continuous variable for the degree of differentiation. Physically, *α* depends on the abundance and sub-cellular localization of proteins and RNAs, as well as other types of signaling and metabolic molecules [45]. The *α*-dynamics of a single cell can be modeled according to a one-dimensional Langevin equation:
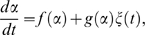
(1)with *f*(*α*) representing the deterministic part of the dynamics and *g*(*α*)*ξ*(*t*) denoting the usual Gaussian white noise term (<*ξ*(*t*)> = 0, <*ξ*(*t*), *ξ*(*t*′)> = *δ*(*t*−*t*′)). In applying Equation (1) one may focus on deterministically dominated (|*f*(*α*)|>*g*(*α*)) or noise modulation-dominated (|*f*(*α*)|<*g*(*α*)) dynamics, both of which can give the same equilibrium distribution of *α*-values when sampled over time. In the following, we concentrate on noise modulation-dominated dynamics. Carrying the predominance of noise to an extreme we completely neglect any deterministic dynamics in our model (*f*(*α*) = 0, corresponding to globally equivalent deterministic potential energy states) and simulate stem cell differentiation as a result of noise modulation alone.

**Figure 1:**
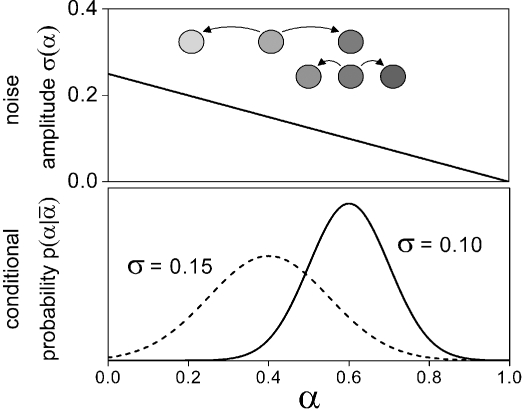
Multiplicative noise-driven dynamics. Upper panel: state-specific noise amplitude (standard deviation) *σ*(*α*) of the Gaussian conditional probability density function (cpdf) 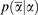 assumed to be a linear decreasing function of *α*. The pictogram shows a cell with *α* = 0.4 being scattered towards *α* = 0.2 and 0.6, respectively (upper row). The subsequent scatter starting at *α* = 0.6 has a smaller range (lower row). This results in an average rightward drift of the peak position of the probability distribution of *α*-values P(*α*) (see also Methods). Lower panel: Gaussian cpdf 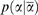 as a function of *α* for 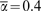 (left) and 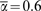 (right). The corresponding standard deviations are *σ*(0.4) = 0.15 and *σ*(0.6) = 0.10.

In order to simulate population dynamics in terms of the number of cells in state *α* we transfer the general ideas of the Langevin approach Equation (1) to a classical population dynamics model which is similar in structure to a master equation for a composite Markov process [46], [47]. The model assumes each cell's *α*-value to randomly fluctuate according to a state-specific noise amplitude *σ*(*α*). Starting from an initial value *α* a cell assumes a new value 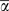 drawn from a Gaussian distribution 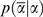 that is centered around *α* and has standard deviation *σ*(*α*) (see Methods). The frequency of this random transfer is determined by the randomization rate *R*(*α*) defining the number of random events per time. We assume *R*(*α*) to increase linearly with the cell proliferation rate *r*(*α*) accounting for cell division as a major source of randomization [48]. Finally, the dynamics of the average number of cells *N*(*α*) in state *α* is governed by the random transfer towards and away from *α*, and by cell proliferation:
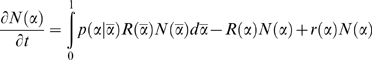
(2)with
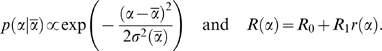
(3)As a consequence of experimental findings [49] we replaced the proliferation term in Equation (2) by the cell cycle model of León et al. [50] assuming cell cycle progression to be a multi-step process (see Methods, Supporting Text S1, and Supporting Figures S1 and S2; five cell cycle steps were used in all simulations). Figure 1 illustrates the general principle of state-specific (multiplicative) noise-driven dynamics. Each cell can gain or loose stem cell properties in a random event. This makes cell differentiation a reversible process in general. A stable stem cell state or terminal differentiation can be introduced by assigning zero noise levels to *α* = 0 or *α* = 1, respectively. Each cell will then finally end up in the respective absorbing state. However, cell proliferation is capable of sustaining a broad population distribution irrespective of individual cell fates.

### Basic assumptions

**Figure 2:**
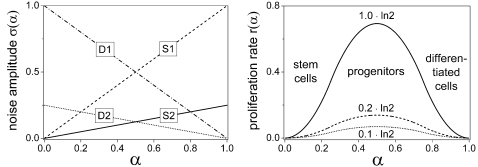
Noise amplitude *σ*(*α*) (left) and proliferation rate *r*(*α*) (right) as a function of cell differentiation *α*. The noise amplitude is shown for four idealized environments: i) two stem cell maintaining environments (S1 and S2) stabilizing stem cell states and ii) two differentiation promoting environments (D1 and D2) stabilizing differentiated states. The proliferation rate is zero (quiescence) at the interval boundaries for pure stem cells and differentiated cells, respectively, and assumes its highest values at intermediate *α*. The maximum proliferation rates *r*_max_ = 0.1, 0.2, and 1.0·ln2/d correspond to minimum cell cycle times of *τ*_min_ = 10, 5, and 1 days, respectively. Note that for exponential growth N(t)∝*e^λ^* ^ln2 t^ = 2*^λ^* ^t^ = 2^t/*τ*^.

Figure 2 shows simplified noise amplitudes *σ*(*α*) and proliferation rates *r*(*α*). The functional form of the noise amplitudes *σ*(*α*) is assumed to be determined by the environment. The stem cell maintaining environments S1 and S2 stabilize stem cell-like states with low *α*-values whereas the differentiation promoting environments D1 and D2 stabilize committed states with large values of *α* by the assignment of low noise levels. The noise amplitudes are assumed to be linear functions of *α* for simplicity. Stem cells and differentiated cells are generally believed to be mostly quiescent whereas progenitors are proliferative. This is reflected by the bell-shaped proliferation rates *r*(*α*) being zero at the interval boundaries and assuming their maximum value *r*_max_>0 halfway in between. However, stem cells and differentiated cells can also be assumed to proliferate in our model. As long as all proliferative states have a positive noise amplitude an initial distribution of *α*-values evolves towards a non-degenerate stationary distribution (see Discussion).

In the following, stem cell populations are charcterized in terms of their numerically calculated relative frequencies 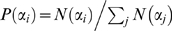 with *N*(*α_i_*) denoting the number of cells in the respective differentiation state interval centered at *α_i_* (see Methods).

### Environmental adaptation

**Figure 3:**
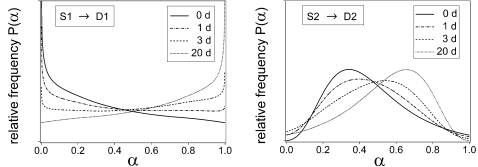
Adaptation dynamics of a stem cell population after instantaneous switching from a stem cell maintaining environment S to a differentiation promoting environment D. Left panel: S1 to D1. Right panel: S2 to D2. Snapshots are taken at the time of switching and 1, 3, and 20 days, respectively, after switching. *R*_0_ = 0.3/d, *R*_1_ = 0.9, *r*_max_ = 1.0·ln2/d.

Figure 3 shows the adaptation dynamics for two cell populations being transferred from a stem cell maintaining environment S to a differentiation promoting environment D. The timescale of the equilibration processes is of the order of days, consistent with experimental data (see below, [24]–[28]). In both cases, the S and D environments fully stabilize pure stem cells (*α* = 0) and differentiated cells (*α* = 1), respectively. However, in the S2 and D2 environments these states can hardly be accessed dynamically because the associated cumulative sum of directed steps is too small on average. This dynamical hindrance together with the stronger stabilization of proliferative progenitor states results in equilibrium distributions that are peaked at intermediate *α*-values. Generally, extensive low noise domains can hardly be accessed from outside these domains.

### Randomization rate

**Figure 4:**
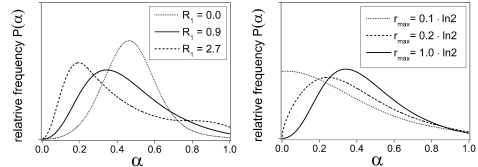
Impact of the noise parameter *R*_1_ (left panel) and the maximum proliferation rate *r*_max_ (right panel) on the stationary distributions for the S2 environment. A high value of *R*_1_ disperses the cells away from the central proliferation zone towards the more noise-reduced states. A small cellular growth as expressed by low values of *r*_max_ lets noise dominate over proliferation even in the presence of a dynamic hindrance in approaching the noise-reduced states (see text). The parameters are identical to those of Figure 3, except for *R*_1_ in the left panel and *r*_max_ in the right panel. The equilibration period was generally 20 days. Systems with small noise amplitudes or low randomization rates may show slow dynamics. For this reason, the equilibration period was set to 60 and 120 days for *r*_max_ = 0.2 and 0.1·ln2/d, respectively. The displayed distributions were checked to be well equilibrated by solving the equilibration condition ∂*N*(*α*)/∂*t* = 0 for *N*(*α*) using Broyden's method [65].

The influence of the noise parameter *R*_1_ on the frequency distribution of *α*-states is illustrated in the left panel of Figure 4 for the S2 environment. A high value of *R*_1_ disperses the cells away from the most proliferating states around the mid-interval towards the noise-reduced states at low *α*-values. The effect of the background noise parameter *R*_0_ is similar but without the state-specific modulation by the proliferation rate *r*(*α*). It drives the cells into the low-noise attractors when proliferation is down-regulated. This is demonstrated in the right panel of Figure 4 for different values of *r*_max_. The equilibrium distribution of non-proliferating cells (*r*_max_ = 0) would be a delta peak at *α* = 0. Conversely, in the absence of noise the population would converge to a delta peak at *α* = 0.5 when starting from an equal distribution. In summary, randomization and proliferation act as antagonists in modulating the cell state distribution, with proliferation enabling the maintenance of subpopulations in environmentally unfavored states.

### Cell differentiation

**Figure 5:**
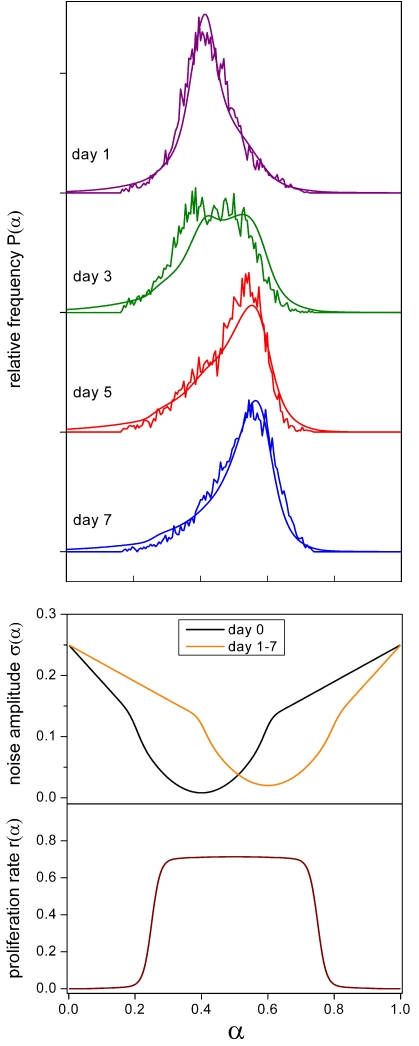
Differentiation dynamics. Fluorescence histograms of CD11b expression in HL60 cells as obtained by flow cytometry at day 1, 3, 5, and 7 of treatment with 0.75% DMSO (upper panel). The experimental data are shown together with the respective simulation results. The model is first equilibrated for a noise amplitude specific for DMSO-free conditions stabilizing CD11b-low-expressing, rather undifferentiated states (lower panel, black curve, day 0). Subsequently, the noise amplitude is instantaneously switched to the DMSO treatment conditions stabilizing CD11b-high-expressing, rather differentiated states (lower panel, orange curve, day 1–7). *R*_0_ = 0.6/d, *R*_1_ = 0.2, *r*_max_ = 1.03·ln2/d. The relative magnitudes of *R*_0_ and *R*_1_ were chosen in order to result in similar differentiation dynamics with and without proliferation as is experimentally suggested for certain HL60 sublines [24], [26], [28].

**Figure 6:**
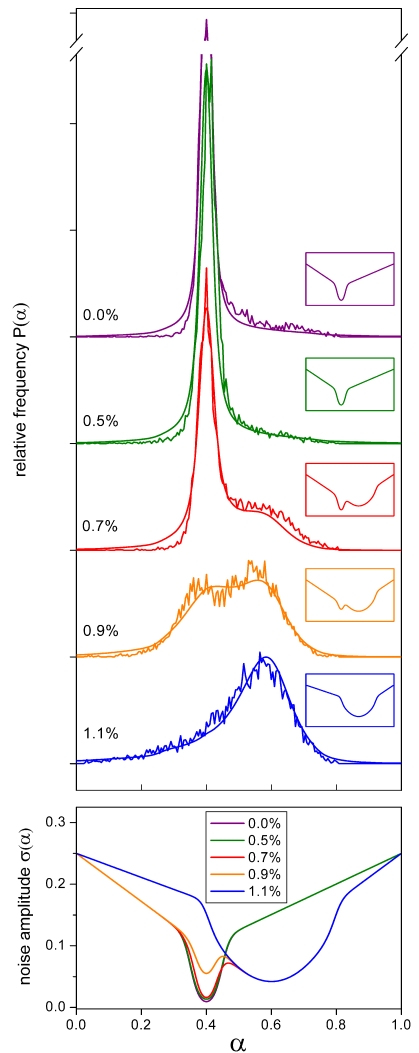
Inducer dose dependence of equilibrated distributions. Fluorescence histograms of CD11b expression in HL60 cells at day 7 of exposure to DMSO doses of 0.0, 0.5, 0.7, 0.9, and 1.1%, respectively (upper panel). The individual noise amplitudes used in the model are shown in the respective insets. They are also pooled in the lower panel (same color). Initially, the model is equilibrated for the environment associated with 0.0% DMSO and low CD11b expression (uppermost curve). After switching to the respective non-zero DMSO conditions that promote high CD11b expression the simulation is continued for a period of 7 days. The DMSO-dependent attractor at *α* = 0.6 is introduced in a switch-like fashion when the DMSO concentration is raised from 0.5 to 0.7%. The original undifferentiated cell attractor at *α* = 0.4 is more gradually eliminated in the higher concentration range of DMSO. *R*_0_ = 0.7/d, *R*_1_ = 0.25, proliferation rate as in Figure 5. The ordinate break cuts the uppermost curve at half peak height.

**Figure 7:**
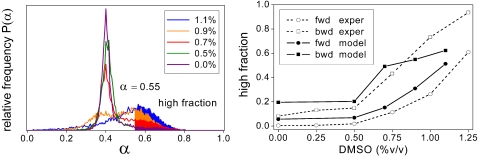
Differentiation-dedifferentiation hysteresis. The left panel illustrates the fraction of CD11b-high-expressing cells (high fraction, filled area, *α*>0.55, corresponding to a relative fluorescence intensity of 645/1024) after 7 days of culture at various DMSO concentrations. The cultures were initiated by untreated HL60 cells. The right panel compares these high fractions (forward direction, fwd, circles) with those for initiation by HL60 cells previously stimulated with DMSO for 7 days (backward direction, bwd, squares). Experimental data, as adapted from [27], are represented by open symbols, model results by filled symbols. DMSO doses used in the pre-culture of the initiating cells: 0% forward direction, 1.1%(model) and 1.25%(experimental) backward direction. Model parameters as in Figure 6.

Recently, we studied the transition of HL60 promyelocytic precursor cells to the neutrophil lineage after stimulation with the inducer dimethyl sulfoxide (DMSO) by monitoring the differentiation marker CD11b (Mac-1) using flow cytometry [27]. The model was applied to two experimental series. In the first series, cells were exposed to 0.75% DMSO for 7 days and monitored for CD11b expression at day 1, 3, 5, and 7 of treatment (Figure 5). In the second series, cells were exposed to DMSO concentrations of 0.0, 0.5, 0.7, 0.9, and 1.1%, respectively, with CD11b expression being measured after 7 days of treatment (Figure 6). For modeling, we mapped the logarithmic fluorescence intensities to the unit interval and identified them with the differentiation state *α*. The functional form of the noise amplitudes as depicted in the lower panel of Figures 5 and 6, respectively, was designed to match the experimental data and provide a proof of principle for our approach (see Methods). The noise amplitude minima constitute attractor states corresponding to the CD11b-low-expressing, rather undifferentiated state (*α* = 0.4) and the CD11b-high-expressing, rather differentiated state (*α* = 0.6), respectively. The assumption of everywhere non-zero noise amplitudes implies reversible (ergodic) *α*-dynamics in agreement with our experimental results (Figure 7) and the finding that certain HL60 sublines have lost the irreversibility of terminal differentiation [25]. The proliferation rate as shown in the lower panel of Figure 5 was chosen to be a hat-like function of *α* implementing a proliferation double-switch (off-on-off). Its width ensures that both the CD11b-low and CD11b-high-expressing cells divide. This is consistent with HL60 cells generally being very proliferative. Moreover, the above assumptions results in the cell population peak positions being largely independent of proliferation, in accordance with the observation that proliferation does not substantially influence HL60 cell differentiation [24], [26], [28]. Lowering of the proliferation rate towards the interval boundaries is required in order to reduce population density tails. This suggests that HL60 cells are almost quiescent for low and high values of *α*. In addition to the above *α*-dependent proliferation rate, we introduced an *α*-independent apoptosis rate increasing linearly with time to account for the experimentally observed decrease in population doubling rates from 1.0 to 0.5/d for 0.0, 0.5, and 0.7% DMSO and from 1.0 to 0.2/d for 0.9 and 1.1% DMSO, respectively (unpublished data, see also Methods). Alternatively, this decrease in proliferation could result from an asymmetric proliferation rate showing a pronounced proliferation maximum for undifferentiated cells (*α* = 0.4). This assumption did, however, not lead to a satisfactory model fit. Nevertheless, a minor asymmetry cannot be completely excluded since small alterations in the proliferation rate can be partly compensated by adjusting the model parameters and the fluorescence data mapping to the unit interval. This weak sensitivity with respect to the proliferation profile is a general feature of our model. The model is most sensitive to the shape of the noise profile at low noise amplitudes. The randomization rates are important for setting the time scale of the differentiation process.

The presented results demonstrate that our model is capable of quantitatively reproducing both the dynamics of the induced differentiation process (Figure 5) and the inducer dose dependence of the respective equilibrated *α*-distributions (Figure 6) using consistent parameter settings. The experimental data agree with the notion of DMSO inducing an attractor state associated with cell differentiation (*α* = 0.6) in a switch-like manner when raising its concentration from 0.5 to 0.7% (Figure 6). An increase in DMSO dosage beyond this point eliminates the original precursor cell attractor (*α* = 0.4) in a more graduated fashion. The position of both attractors neither depends on time nor on DMSO dosage suggesting that DMSO activates a single noise reduction mechanism.

### Differentiation-dedifferentiation hysteresis

The experimental system exhibits hysteresis in that the final distribution arrived at after 7 days of culture depends on the distribution of the initial cell population. In Figure 7 the final distribution at various DMSO doses is characterized by the fraction of cells that show a high CD11b expression. This high fraction is lower for the culture being initiated by untreated HL60 cells (forward direction) compared to HL60 cells previously treated with a high DMSO dosage for 7 days (backward direction). Stochastic systems, like the one presented here, generally evolve towards unique equilibrium distributions but may exhibit a kinetic hysteresis [47]. Figure 7 (right panel) shows the experimental and simulated high fractions (*α*>0.55) demonstrating that for the same parameter settings as in Figure 6 the kinetic hysteresis displayed by our model is consistent with the experimental observations.

### Population regeneration

**Figure 8:**
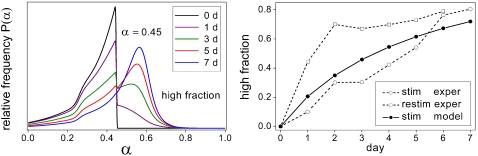
Population regeneration. First, cell population dynamics were simulated for a dosage of 0.75% DMSO and a period of 7 days (compare Figure 5) starting from an untreated cell distribution additionally sorted for low CD11b expression (*α*≤0.45, corresponding to a relative fluorescence intensity of 423/1024). Subsequently, the final distribution obtained after 7 days of culture was also sorted for CD11b-low-expressing cells and exposed to the same DMSO dosage for another 7 days (left panel, snapshots at 0, 1, 3, 5, and 7 days). The right panel shows the time course of the CD11b-high-expressing fraction (high fraction, *α*>0.45) for the primary stimulation (circles) and the restimulation (squares). Experimental data, as adapted from [27], relate to 0.8% DMSO and are represented by open symbols. Model results are shown as filled symbols. The model restimulation curve (not shown) is quasi-identical to the stimulation curve.

In addition to the cell differentiation assays of the previous paragraphs we performed population regeneration (restimulation) experiments [27]. In a first experimental step cells were stimulated with 0.8% DMSO for 7 days and FACS-sorted for cells with a low CD11b expression. Subsequently, these CD11b-low-expressing cells were restimulated with the same DMSO dosage for another 7 days. We simulated FACS-sorting by retaining the fraction of cells with *α*≤0.45 in the distributions obtained after 7 days of 0.75% DMSO treatment corresponding to the results of Figure 5. The system was then further evolved for 7 days using the same DMSO dosage conditions (Figure 8, left panel). The right panel of Figure 8 displays the fraction of CD11b-high-expressing cells (high fraction, *α*>0.45) during stimulation and restimulation. While the experimental data of the primary stimulation agree quite well with the model results the cellular response to restimulation is much faster than predicted by our present one-dimensional model (priming effect, see Discussion).

## Discussion

Noise is ubiquitous in biological systems and must be controlled to ensure reliable cell functioning, at least in higher multicellular organisms that feature noise-sensitive processes like alternative splicing and epigenetic regulation of gene expression. Noise regulation is most economic if applied only to those cellular states that are relevant under the prevailing environmental conditions. Noise regulation is thus expected to depend on the match between the internal state of a cell and its environment. In the present study we introduced a simple few-parameter model of stem cell and progenitor population dynamics that is explicitly based on noise regulation. Applying this model to a recent unique data set measuring various aspects of the dynamics and inducer dose dependence of stimulated differentiation in promyelocytic precursor cells [27] we demonstrate that our approach provides a consistent description of these data including differentiation-dedifferentiation hysteresis as well as population regeneration. Our results suggest that stem cell and progenitor population dynamics can be effectively driven by state-specific noise. These findings provide new insights into phenomena like stem cell maintenance, plasticity, and environmental adaptation.

The model assumes that cell population dynamics result from random fluctuations of the state of differentiation *α*. In the present study we simply identified *α* with the logarithmic CD11b marker expression. More generally, measuring a number of stem cell, differentiation or lineage markers, like promylelocytic or granulocyte markers in hematopoiesis, would enable a definition of the state of differentiation as a multivariate function of the measured markers. Clearly, changes in *α* correspond to changes in the combined marker expression, and thus to transitions between complex regulatory network activation patterns. In our present model these transitions are understood as random fluctuations caused by molecular level noise inherent in biological systems. Biological noise was proposed to arise from chromatin remodeling, promoter activation, and transcription [11], [31], [51]–[55]. Moreover, the spatial variation of molecular concentrations as well as asymmetric partitioning of proteins during cell division can be considered further sources of noise [45], [48], [56]. It has been proposed that biological systems have built-in molecular devices for noise control [7]–[12]. These control mechanisms are of specific importance in developing organisms with the Wnt signaling pathway as a prominent example [11], [13]. Further support comes from recent experimental findings demonstrating that noise is down-regulated in embryonic stem cells [14], [15]. The present modeling concept assumes that state-specific noise regulation in response to environmental signals serves as a selector of certain differentiation states representing specific functional cellular programs. This noise-driven selection scheme appears to be an economic general purpose mechanism for environmental adaptation and diversification since only the selected cell states need to be noise-reduced. Stem cell differentiation in higher organisms is controlled by epigenetic phenomena. Prominent mediators of epigenetic reprogramming are Polycomb Group proteins [57]. Their expression level has been shown to be modulated by environmental inputs, thus linking extracellular cues to reprogramming of the epigenome [58]. Together with the fact that epigenetic gene silencing has a strong stochastic component [40], [41] this suggests environmentally regulated epigenetic processes as effectors of noise regulation during differentiation.

The results of the present study demonstrate the capability of our model to explain the dynamics of differentiation marker expression in response to variable doses of soluble inducing factors as well as the regeneration of cell populations from subpopulations. Population structure and dynamics were most sensitive to the shape of the noise profile at low noise amplitudes. Measured proliferation rates were consistently described. Chang et al. [27] observed that the regeneration from subpopulations under DMSO treatment was faster for pre-stimulated cells compared to native cells with the same marker expression. Our present one-dimensional model cannot describe these priming effects, thus calling for model extensions that account for multi-stable and multi-step differentiation processes in a multidimensional system as suggested in [27]. Recent results suggest that linage choice in hematopoietic progenitor cells is indeed a multi-step process that is noise controlled [59]. The same work also demonstrates regeneration of whole populations from various subpopulations in hematopoietic systems. This supports the assumption of reversible cell differentiation underlying the present study. Simulating cell differentiation as a result of noise modulation alone is another abstraction specific to our model. In general, the deterministic part of the dynamics (f(a) in the Langevin equation (1)) will be different from zero. As a consequence, deterministic system behavior, as commonly modeled by chemical rate equations, will prevail in low noise states enabling more reliable cell functioning. This implication was already noted for models with constant noise amplitude [43].

The present model has been developed in particular for simulating the dynamics of stem cell and progenitor adaptation to different environments. Stem cell niches are assumed to reduce state fluctuations of stem cell-like states. Thus, the progeny of stem cells remains stem cell-like. This mimics symmetric cell division maintaining or expanding the stem cell pool. Transfer of stem cell populations into environments that promote differentiation leads to destabilization of stem cell-like states. This process can be understood as stem cell activation. In direct analogy to the destabilizing effect of noise assumed in the present study, fluctuations of Nanog, a potent stem cell regulator, were recently suggested to open temporal windows for the initiation of differentiation processes [16]. The progeny of cells in high-fluctuation states quickly diverges. This mimics asymmetric cell division. Such destabilized cells adapt to their environment by traveling towards low-fluctuation states. For near zero noise amplitudes the cells become trapped in these states for their life time rendering this adaptation process effectively irreversible.

According to our model high-fluctuation states can be kept populated by cell proliferation. Vice versa, a population-wide proliferation stop would accumulate a maximum number of cells in low-noise states. Due to the prevalence of deterministic dynamics over noise in these states, the accumulated cells should function more predictably. However, the resulting homogeneous cell population of highly specialized individuals will in general be less flexible for adaptation to unexpected environmental changes [2]. Repeated exposure to the same environmental changes could have resulted in the evolution of efficient deterministic adaptation mechanisms that do not rely on noise regulation. Such mechanisms are most likely found in simple organisms like the lactose and tryptophan utilization networks in *E.coli* and other bacteria [60]–[62]. However, Kashiwagi et al. [34] showed that *E.coli* cells equipped with a synthetic bistable gene switch actively select the attractor state that allows survival in one of two alternative nutritional environments. Through promoter swap experiments they demonstrated that this selection mechanism is not hard-wired in the *E.coli* genome, and thus must be non-deterministic and driven by gene expression noise.

The transfer of cells from a stem cell niche to an *in vitro* culture constitutes a drastic change in environmental conditions leading to a complete reorganization of the stem cell population. Similar effects are expected for the recruitment of *in vivo* stem cells to implanted biomedical scaffolds. A better understanding of such adaptation processes will be essential for the optimization of stem cell culture protocols and the design of injectable biomaterials [22], [63], [64].

Subsequent to the present studies, follow-up experiments addressing the imprinting influence of the environment on cellular noise regulation and the reversibility of stem cell development must effectively quantify noise during cellular differentiation and dedifferentiation. Future projects should thus analyze the fluctuations of stem cell marker expression in single cells while measuring their proliferation activity at the same time. This can be achieved by automated long-term cell tracking which enables the reconstruction of cellular pedigrees in high-throughput studies. Such studies are expected to uncover well defined cellular states with significantly reduced or amplified noise levels. Moreover, they are prerequisite in the detection of signaling pathways acting as noise control devices.

In conclusion, we suggest that noise regulation can be effective in cellular development and environmental adaptation. It is expected to be relevant especially in higher multicellular organisms that comprise exposed noise-sensitive phenomena. Decoding the ‘noise landscape’ will be essential for the understanding of cell fate control and development.

## Methods

### Experimental Methods

#### Cell Culture and Differentiation

HL-60 cells (ATCC) were cultured in IMDM medium (ATCC) supplemented with 10% fetal bovine serum and 1% glutamine plus penicillin and streptomycin. Cells of passage 7 (after receipt from ATCC) at a density of 1.0·10^6^ cells/ml and growing at a basal rate of 1.3–1.7 day ^−1^ were treated with variable concentrations of DMSO (Sigma) ranging from 0.3% to 1.25% (v/v) to induce differentiation. At each time point, cells were harvested from the suspension culture, pelleted, and processed for flow cytometry analysis.

#### Flow cytometry and Fluorescence Activated Cell Sorting (FACS)

For the Guava- PCA system (see below) 200,000 cells were pelleted and incubated in 7 µl of CD11b/MAC-I R-PE conjugated fluorescence antibody (BD Pharmingen) on ice for 30 min, washed with ice-cold 1% fetal calf serum/PBS/0.01% NaN_3_ (NaN_3_ is left out in sorting experiments), and resuspended in the same buffer at 10^6^ cells/ml density for analysis. For fluorescence-activated cell sorting, staining was scaled up 10-fold to 50 µl of CD11b/MAC-I R-PE conjugated fluorescence antibody (BD Pharmingen) per 10^6^ cells and cells were resuspended at 8–10·10^6^ cells/ml. Flow cytometry was performed on a Guava-PCA microfluidic-based flow cytometer (GuavaTechnologies, Inc). Fluorescence activated cell sorting was performed with either a Becton Dickinson FACSVantage (Becton Dickinson) or a Becton Dickinson FACSAria (Becton Dickinson) flow cytometer. Data analysis was done with either CytoSoft™ 2.1.1. (GuavaTechnologies, Inc) or WinMDI software. For cell sorting, starting cell number ranged between 40–80·10^6^ cells, and cells were sorted into ice-cold medium for a maximum of 3 hours. Gates for sorting the CD11b-low-expressing subpopulation in the 0.8% DMSO-treated samples were set relative to an untreated, native population. The latter was also mock sorted and processed in exactly the same way as the former to control for the effects of FACS sorting on cellular expression of CD11b. To remove the staining antibody before reculturing, pelleted cells were suspended in pH. 2.25 MES (morpholinoethanesulfonic acid)/Tris buffer for 30 s. A 10-fold volume of pH 7.4 PBS was immediately added for neutralization and the cells were pelleted and resuspended in culture medium. After antibody removal the cells had fluorescence signal intensities on par with unstained HL60 cells and exhibited normal viability for future immunofluorescence staining. For detailed Methods see [27].

### Theoretical Methods

The present modeling approach is based on the dynamics of stem cell populations stratified with respect to cell differentiation. Cell differentiation is defined through the variable *α*∈[0,1] with *α* = 0 for pure stem cells and *α* = 1 for fully differentiated cells. The model assumes cell differentiation to be subject to random changes defined by the conditional probability density function (cpdf) 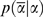 for 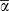 given *α* and the randomization rate *R*(*α*) quantifying the number of random events per time (Equations (2) and (3)). The cpdf 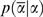 is assumed to be Gaussian centered at *α* with standard deviation (noise amplitude) *σ*(*α*). It is renormalized to unity for each *α* to account for the truncation to the interval [0,1]. The noise amplitude 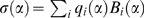 is specified as a sum of piecewise linear or quadratic functions *q_i_*(*α*) = *u*_0_+*u*_1_(*α*−*α_iq_*)+*u*_2_(*α*−*α_iq_*)^2^ localized by tanh-type radial basis functions 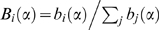 with *b_i_*(*α*) = 1/2 tanh[(*r_i_*−*α*+*α_ib_*)/*s_i_*]+1/2 tanh[(*r_i_*+*α*−*α_ib_*)/*s_i_*], in which *α_iq_* and *α_ib_* denote the offset of the polynomial and the radial base, respectively, whereas *r_i_* specifies the characteristic radius, and *s_i_* the transfer width of the base. Cells are assumed to proliferate according to the growth rate *r*(*α*) and the time-dependent apoptosis rate *a*(*t*) = *a*_1_ *t* irrespective of generation. The two-dimensional rate equation for the average number of cells is numerically solved by the explicit Euler Forward Method on a 2D-grid of discrete differentiation values *α_i_*, *i* = 1,…,*n_a_*, and generation-specific cell cycle phases *k* = 1,…,*n_p_* according to
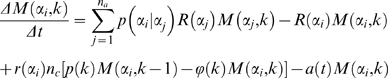
(4)in which *n_c_* denotes the number of cell cycle phases per generation, *ρ*(*k*) = 2 if *k*≡1(mod *n_c_*) to account for cell doubling and *ρ*(*k*) = 1 otherwise. Furthermore, *ϕ*(*k*) = 0 if *k* = *n_p_* and *ϕ*(*k*) = 1 otherwise. It is understood that *M*(*a_i_*, *k*−1) = 0 for *k* = 1. The cell cycle terms in the second row of Equation (4) implement the continuous cell cycle model of León et al. [50] without *G*_0_ phase arrest. The number of cells in generation *l* is calculated by summing over its cell cycle phases 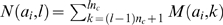. The dynamics of the marginal relative frequencies associated with cell differentiation can easily be derived from 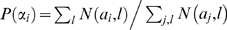.

Truncation of the cpdf 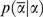 to the unit interval generally results in non-symmetric scattering and thus in a non-vanishing drift term *A*(*α*) as defined for the Fokker-Planck equation [47]. This term mimics a deterministic dynamic component corresponding to *f*(*α*) in the Langevin equation (1). The results of the present study were checked against either using the numerically determined non-zero *A*(*α*) or setting *A*(*α*) = 0 in the equilibrated distributions. We found no notable difference except for the S1 to D1 transition shown in Figure 3, for which, however, also the Fokker-Planck approximation to the master equation breaks down.

## Supporting Information

Text S1 (0.03 MB DOC)

Figure S1 Cell generation distribution. Experimental data (large open circles) as adapted from Holtz et al. [1] and model results obtained by assuming a number of n_c_ = 1 (filled squares) and 5 (filled circles) cell cycle steps in the proliferation model of León et al. [2]. Assuming only one cell cycle step corresponding to Equation (2) of the MM is not in agreement with the experimental data. Model parameters as in Figure 3, left panel, of the MM except for r_max_ = 1.1×ln2/d. CD34+/CD38- FACS-sorting was simulated by retaining cells with α≤0.17 resulting in a percentage of 23% primitive progenitors. The distribution obtained by retaining all cells in the simulation (open circles) illustrates that the primitive progenitor fraction is initially depleted for fast proliferating cells.
(0.37 MB TIF)

Figure S2 Number of cell cycle steps. Equilibrium distributions of α-values corresponding to Figure 4, right panel, of the MM for a number of cell cycle steps n_c_ = 1, 2, 5, and 20. The larger the number of cell cycle steps n_c_ the smaller the effective proliferation rate (compare also Figure S1) driving the cells into the most attractive state at α = 0. The effect of n_c_ on the α-dynamics saturates for high values of n_c_ and is smaller for higher proliferation rates.
(0.29 MB TIF)

## References

[1] Korobkova E, Emonet T, Vilar JM, Shimizu TS, Cluzel P (2004). From molecular noise to behavioural variability in a single bacterium.. Nature.

[2] Thattai M, van Oudenaarden A (2004). Stochastic gene expression in fluctuating environments.. Genetics.

[3] Samoilov M, Plyasunov S, Arkin AP (2005). Stochastic amplification and signaling in enzymatic futile cycles through noise-induced bistability with oscillations.. Proc Natl Acad Sci U S A.

[4] Fange D, Elf J (2006). Noise-induced Min phenotypes in E. coli.. PLoS Comput Biol.

[5] Maamar H, Raj A, Dubnau D (2007). Noise in gene expression determines cell fate in Bacillus subtilis.. Science.

[6] Samoilov MS, Price G, Arkin AP (2006). From fluctuations to phenotypes: the physiology of noise.. Sci STKE.

[7] Thattai M, van Oudenaarden A (2002). Attenuation of noise in ultrasensitive signaling cascades.. Biophys J.

[8] Stelling J, Sauer U, Szallasi Z, Doyler FJ, Doyle J (2004). Robustness of cellular functions.. Cell.

[9] Acar M, Becskei A, van Oudenaarden A (2005). Enhancement of cellular memory by reducing stochastic transitions.. Nature.

[10] Hooshangi S, Thiberge S, Weiss R (2005). Ultrasensitivity and noise propagation in a synthetic transcriptional cascade.. Proc Natl Acad Sci U S A.

[11] Arias AM, Hayward P (2006). Filtering transcriptional noise during development: concepts and mechanisms.. Nat Rev Genet.

[12] El-Samad H, Khammash M (2006). Regulated degradation is a mechanism for suppressing stochastic fluctuations in gene regulatory networks.. Biophys J.

[13] Freeman M (2000). Feedback control of intercellular signalling in development.. Nature.

[14] Szutorisz H, Georgiou A, Tora L, Dillon N (2006). The proteasome restricts permissive transcription at tissue-specific gene loci in embryonic stem cells.. Cell.

[15] Zwaka TP (2006). Keeping the noise down in ES cells.. Cell.

[16] Chambers I, Silva J, Colby D, Nichols J, Nijmeijer B (2007). Nanog safeguards pluripotency and mediates germline development.. Nature.

[17] Potten CS, Loeffler M (1990). Stem cells: attributes, cycles, spirals, pitfalls and uncertainties. Lessons for and from the crypt.. Development.

[18] Loeffler M, Roeder I (2002). Tissue stem cells: definition, plasticity, heterogeneity, self-organization and models–a conceptual approach.. Cells Tissues Organs.

[19] Wilson A, Trumpp A (2006). Bone-marrow haematopoietic-stem-cell niches.. Nat Rev Immunol.

[20] Grayson WL, Zhao F, Izadpanah R, Bunnell B, Ma T (2006). Effects of hypoxia on human mesenchymal stem cell expansion and plasticity in 3D constructs.. J Cell Physiol.

[21] Engler AJ, Sen S, Sweeney HL, Discher DE (2006). Matrix elasticity directs stem cell lineage specification.. Cell.

[22] Ingber DE (2006). Mechanical control of tissue morphogenesis during embryological development.. Int J Dev Biol.

[23] Gosh K, Ingber DE (2007). Micromechanical control of cell and tissue development: Implications for tissue engineering.. Adv Drug Deliv Rev.

[24] Drayson MT, Michell RH, Durham J, Brown G (2001). Cell proliferation and CD11b expression are controlled independently during HL60 cell differentiation initiated by 1,25 alpha-dihydroxyvitamin D(3) or all-trans-retinoic acid.. Exp Cell Res.

[25] Kitajima K, Haque M, Nakamura H, Hirano T, Utiyama H (2001). Loss of irreversibility of granulocytic differentiation induced by dimethyl sulfoxide in HL-60 sublines with a homogeneously staining region.. Biochem Biophys Res Commun.

[26] Brown G, Drayson MT, Durham J, Toellner KM, Hughes PJ (2002). HL60 cells halted in G1 or S phase differentiate normally.. Exp Cell Res.

[27] Chang HH, Oh PY, Ingber DE, Huang S (2006). Multistable and multistep dynamics in neutrophil differentiation.. BMC Cell Biol.

[28] Gibbs JD, Liebermann DA, Hoffman B (2007). Terminal myeloid differentiation is uncoupled from cell cycle arrest.. Cell Cycle.

[29] Isaacs FJ, Hasty J, Cantor CR, Collins JJ (2003). Prediction and measurement of an autoregulatory genetic module.. Proc Natl Acad Sci U S A.

[30] Huang S, Eichler G, Bar-Yam Y, Ingber DE (2005). Cell fates as high-dimensional attractor states of a complex gene regulatory network.. Phys Rev Lett.

[31] Kaern M, Elston TC, Blake WJ, Collins JJ (2005). Stochasticity in gene expression: from theories to phenotypes.. Nat Rev Genet.

[32] Quesenberry PJ, Dooner G, Dooner M, Colvin G (2005). The stem cell continuum: considerations on the heterogeneity and plasticity of marrow stem cells.. Stem Cell Rev.

[33] Viswanathan S, Davey RE, Cheng D, Raghu RC, Lauffenburger DA (2005). Clonal evolution of stem and differentiated cells can be predicted by integrating cell-intrinsic and -extrinsic parameters.. Biotechnol Appl Biochem.

[34] Kashiwagi A, Urabe I, Kaneko K, Yomo T (2006). Adaptive response of a gene network to environmental changes by fitness-induced attractor selection.. PLoS ONE.

[35] Roeder I, Horn M, Glauche I, Hochhaus A, Mueller MC (2006). Dynamic modeling of imatinib-treated chronic myeloid leukemia: functional insights and clinical implications.. Nat Med.

[36] Roeder I, Lorenz R (2006). Asymmetry of stem cell fate and the potential impact of the niche: observations, simulations, and interpretations.. Stem Cell Rev.

[37] Loeffler M, Roeder I (2004). Conceptual models to understand tissue stem cell organization.. Curr Opin Hematol.

[38] Roeder I, Braesel K, Lorenz R, Loeffler M (2007). Stem cell fate analysis revisited: interpretation of individual clone dynamics in the light of a new paradigm of stem cell organization.. J Biomed Biotechnol.

[39] Glauche I, Cross M, Loeffler M, Roeder I (2007). Lineage specification of hematopoietic stem cells: mathematical modeling and biological implications.. Stem Cells.

[40] Rando OJ, Paulsson J (2006). Noisy silencing of chromatin.. Dev Cell.

[41] Xu EY, Zawadzki KA, Broach JR (2006). Single-cell observations reveal intermediate transcriptional silencing states.. Mol Cell.

[42] Waddington CH (1956). Principles of Embryology.

[43] Wang J, Huang B, Xia X, Sun Z (2006). Funneled landscape leads to robustness of cell networks: yeast cell cycle.. PLoS Comput Biol.

[44] Ao P, Kwon C, Qian H (2007). On the existence of potential landscape in the evolution of complex systems.. Complexity.

[45] Lecuyer E, Yoshida H, Parthasarathy N, Alm C, Babak T (2007). Global analysis of mRNA localization reveals a prominent role in organizing cellular architecture and function.. Cell.

[46] Kepler TB, Elston TC (2001). Stochasticity in transcriptional regulation: origins, consequences, and mathematical representations.. Biophys J.

[47] van Kampen NG (2004). Stochastic processes in physics and chemistry.

[48] Beckmann J, Scheitza S, Wernet P, Fischer JC, Giebel B (2007). Asymmetric cell division within the human hematopoietic stem and progenitor cell compartment: identification of asymmetrically segregating proteins.. Blood.

[49] Holtz MS, Slovak ML, Zhang F, Sawyers CL, Forman SJ (2002). Imatinib mesylate (STI571) inhibits growth of primitive malignant progenitors in chronic myelogenous leukemia through reversal of abnormally increased proliferation.. Blood.

[50] Leon K, Faro J, Carneiro J (2004). A general mathematical framework to model generation structure in a population of asynchronously dividing cells.. J Theor Biol.

[51] Becskei A, Kaufmann BB, van Oudenaarden A (2005). Contributions of low molecule number and chromosomal positioning to stochastic gene expression.. Nat Genet.

[52] Pedraza JM, van Oudenaarden A (2005). Noise propagation in gene networks.. Science.

[53] Raser JM, O'Shea EK (2005). Noise in gene expression: origins, consequences, and control.. Science.

[54] Bar-Even A, Paulsson J, Maheshri N, Carmi M, O'Shea E (2006). Noise in protein expression scales with natural protein abundance.. Nat Genet.

[55] Newman JR, Ghaemmaghami S, Ihmels J, Breslow DK, Noble M (2006). Single-cell proteomic analysis of S. cerevisiae reveals the architecture of biological noise.. Nature.

[56] Kerszberg M (2004). Noise, delays, robustness, canalization and all that.. Curr Opin Genet Dev.

[57] Schwartz YB, Pirrotta V (2007). Polycomb silencing mechanisms and the management of genomic programmes.. Nat Rev Genet.

[58] De Santa F, Totaro MG, Prosperini E, Notarbartolo S, Testa G (2007). The histone H3 lysine-27 demethylase Jmjd3 links inflammation to inhibition of polycomb-mediated gene silencing.. Cell.

[59] Chang HH, Hemberg M, Barahona M, Ingber DE, Huang S (2008). Transcriptome-wide noise controls lineage choice in mammalian progenitor cells.. Nature.

[60] Ozbudak EM, Thattai M, Lim HN, Shraiman BI, Van Oudenaarden A (2004). Multistability in the lactose utilization network of Escherichia coli.. Nature.

[61] Tabaka M, Cybulski O, Holyst R (2008). Accurate genetic switch in Escherichia coli: novel mechanism of regulation by co-repressor.. J Mol Biol.

[62] Merino E, Jensen RA, Yanofsky C (2008). Evolution of bacterial trp operons and their regulation.. Curr Opin Microbiol.

[63] Gosh K, Ingber DE (2007). Micromechanical control of cell and tissue development: Implications for tissue engineering.

[64] Ingber DE, Levin M (2007). What lies at the interface of regenerative medicine and developmental biology?. Development.

[65] Press WH, Flannery BP, Teukolsky SA, Vetterling WT (1992). Numerical Recipes in C.

