# Supplementary material for: Noise-Driven Stem Cell and Progenitor Population Dynamics
Source: PLoS One. 2008 Aug 13;3(8):e2922. doi: 10.1371/journal.pone.0002922 (PMC2488392; doi:10.1371/journal.pone.0002922)
Supplement: Text S1 — (0.03 MB DOC) [file pone.0002922.s001.doc]

## Supporting Information

## Cell proliferation model

The application of our model to the experimental data on the proliferation of hematopoietic progenitor cells published by Holtz et al. [1] suggested the replacement of the proliferation term in Equation (2) of the main manuscript (MM) by the cell cycle model of León et al. [2] that assumes cell cycle progression to be a multi-step process. The experiments of Holtz et al. [1] can briefly be described as follows. CD34+ progenitors were first FACS-sorted for a fraction of CD34+/CD38- primitive progenitors. These were subsequently cultivated for 4 d in a proliferation assay and finally analyzed for their cell generation distribution using carboxyfluoroscein succinimidyl ester (CFSE) labeling. As shown in Figure S1 our model was able to reproduce the generation distribution assuming the environment S1 to environment D1 transition (Figure 2 of the MM, left panel, parameters as given in the caption of this figure except for $r_{\max}=$1.1 ∙ ln2/d), a number of cell cycle steps $n_{c}=$ 5 (generally used throughout the present study), and a percentage of 23% FACS-sorted primitive progenitors, well in the range of the experimental fraction graphically indicated in Figure 6 of [1].

Error: Reference source not foundError: Reference source not foundThe influence of the number of cell cycle steps $n_{c}$ on the distribution of $\alpha$-values is demonstrated in Figure S2. Generally, higher values of $n_{c}$ correspond to lower effective proliferation rates resulting in a higher relative importance of the noise-driven dynamics.

**References**

1. Holtz MS, Slovak ML, Zhang F, Sawyers CL, Forman SJ, et al. (2002) Imatinib mesylate (STI571) inhibits growth of primitive malignant progenitors in chronic myelogenous leukemia through reversal of abnormally increased proliferation. Blood 99: 3792-3800.

2. Leon K, Faro J, Carneiro J (2004) A general mathematical framework to model generation structure in a population of asynchronously dividing cells. J Theor Biol 229: 455-476.
